# Comparing Apples to Apples: Validity Evidence for an Innovative Practical Trials Approach to Evaluating the Effectiveness of Medical Curricula

**DOI:** 10.1007/s40670-025-02577-4

**Published:** 2025-11-28

**Authors:** Jeffrey J. H. Cheung, Aftab Merchant

**Affiliations:** 1 Department of Medical Education, University of Illinois College of Medicine, 808 S. Wood St., 966 CMET MC 591, Chicago, IL 60612 USA; 2 Department of Medical Education, Kirk Kerkorian School of Medicine, University of Nevada Las Vegas, Las Vegas, NV USA

**Keywords:** Curricular evaluation, Non-inferiority, Practical trials, Validity evidence, Assessment, Medical education

## Abstract

**Background:**

Evaluating curricular changes in medical education remains challenging due to their complexity. Traditional pre-post comparisons can lack sufficient validity evidence, which can result in flawed interpretations and decision-making. This study proposes an innovative approach using practical trials and non-inferiority designs to strengthen the validity of curricular evaluations.

**Methods:**

We conducted a non-inferiority study comparing medical students’ exam performance between two large cohorts (n_1_ = 304; n_2_ = 294) before and after a curricular change that significantly reduced in-person anatomy lab time. We explore how the results and conclusions may vary based on whether cohort differences in exam difficulty (using faculty-determined Angoff scores) and student ability (using MCAT and GPA data) are accounted for, and what a priori non-inferiority margin is used.

**Results:**

Raw exam scores showed no significant difference between cohorts (Mean difference = 0.13%). However, after adjusting for exam difficulty, students in the new curriculum scored 4.97% lower, *p* < 0.001. Adjusting for student ability further reduced this difference to 4.27%, *p* < 0.001. Using the 99% confidence interval for our mean differences and an a priori non-inferiority margin of 10%, we determined the two curricula were non-inferior.

**Discussion:**

This study demonstrates a novel approach to evaluating curricular changes. While we concluded that reduced anatomy time did not meaningfully decrease student performance, our analyses also reveal how various interpretations can arise based on the approach to comparison. We offer guidance on how practical trials can be combined with strategies to account for cohort differences to enhance the validity of curricular comparisons.

## Introduction

Curricular reform in medical education is often driven by the need to improve student learning, enhance training, and address evolving accreditation standards. However, despite the substantial investments in curricular modifications, there remains a critical challenge in determining whether these changes yield meaningful improvements in educational outcomes due to threats to the validity of assessments and comparison methods. Given the high stakes of curricular decision-making, there is a pressing need for methodologies that can provide a transparent and more valid understanding of whether curricular innovations truly benefit students. This study addresses this gap by proposing an alternative framework for curricular evaluation that strengthens the validity of comparisons and enhances the ability of educators and researchers to make evidence-based decisions.

A common strategy used to gauge the learning effectiveness of curricular change is to compare different groups of students’ performance on a summative exam score, before and after the curricular change. A number of variables can be used for these comparisons, including performance on national exams (e.g., USMLE), internal program metrics (e.g., admissions or matching data), learning environment and mistreatment data, student performance (e.g., grades or exam scores), and internal evaluations (e.g., course evaluations) [1]. These data are then used to justify or overturn a curricular change. This approach, however, introduces numerous threats to the validity of these interpretations, which warrants improved methodologies and assessment strategies to bolster curricular decision-making. Without strong validity evidence, curricular comparisons can lead to misleading conclusions that go on to negatively shape educational policy and faculty investment. In this research report, we provide an overview of these challenges and offer a novel approach to curricular evaluation that aims to improve the validity of these measures and ultimately support the decision-making of those responsible for evaluating curricular changes.

### Challenges of Effective Curricular Comparison

Whereas educational studies can use controlled experiments to compare new innovations with standard educational practice, studies of curricular change do not have the luxury of randomization and direct comparison. It would be unethical for two groups of students at a medical school to be randomized into two vastly different curricula. Because of this, curricula are often evaluated by comparing student learning outcomes across cohorts of students from before and after a curricular change.

When evaluating the impact of curricular change, cohort comparisons may be more feasible, simple, and convenient to use; they also introduce several limitations that threaten their validity. First, educators must discern whether any detected differences between cohorts are of practical significance. Because cohort comparisons often involve large sample sizes from dozens or possibly hundreds of students, this can doom many comparisons to statistical significance [2, 3]. Curriculum assessment data comes from many sources, including student quizzes and exam scores, evaluations from clerkship instructors, data on whether students completed course evaluations, surveys of student engagement, learning environment measures etc. These data sources and sheer number of data points present a plethora of potential comparisons an educator can make, further increasing the likelihood of statistical significance. Further challenging the validity of cohort comparisons is the need for alignment between the construct(s) a curricular innovation or change was intended to address and the selected measures. For example, a curricular innovation with the goal of improving student engagement would require different assessments than one with the goal of improving students’ anatomy knowledge. And even assuming appropriate alignment between our constructs of interest and assessments and uncover a statistically significant difference, this difference may not be of practical significance. Practical significance requires that the effect is large enough to be of relevance to key stakeholders, and the associated costs associated with a curricular innovation (e.g., greater faculty resources or educational technology) are acceptable.

Another threat to validity is the durability of the effects from a curricular change. The detectable effects of a curricular innovation may be transient artefacts, due to temporary increases in faculty and student motivation and engagement that accompanies any curricular change [4]. That is, the effectiveness of the intervention is not the reason why a difference between cohorts is detected, but rather the fact that there is a change to begin with. Finally, effective comparison of cohorts is affected by the differences in measures and of the cohorts themselves. Even when selecting the appropriate measures there may still be relevant cohort differences that affect our interpretation of any comparison. This includes potential cohort differences between the assessments (e.g., content or item difficulty) or between students (e.g., demographics or academic ability) that can affect the interpretation of the results.

### Applying Practical Trials to Understand Curricular Change

Generating meaningful insights from curricular evaluations is challenging. Large scale curricular comparisons have largely yielded mixed or non-existent effects [5–7], which has raised concerns about the value of curricular change. Instead, program evaluation researchers have suggested scholars move beyond questioning “did it work?” to evaluating more nuanced questions pertaining to how, why, and what else happened as part of a curricular change [8]. Similarly, proponents of theory-driven research argue for greater control and theoretical comparisons that generate generalizable understanding of how to improve health professions education [9–11]. Despite strong arguments for more holistic nuanced program evaluation and theory-driven research, there remains a critical and practical need to compare student performance between curricula: ensuring these changes do not hamper student learning. While other measures of student wellness and satisfaction are also critical; educators, curriculum leaders, and citizens invested in the development of future healthcare professionals must first and foremost ensure that any changes to curriculum do not negatively affect the clinical expertise that trainees develop.

One research methodology that may support educators in drawing more valid inferences about the effectiveness of curricular modifications are practical trials [12]. Practical trials refer to research that aims to clarify how new innovations compare with existing alternatives in terms of effectiveness and cost.^2^ Rather than test or build educational theory using highly controlled interventions (controlling all variables but a single dependent variable), practical trials have less strict criteria that sacrifices internal validity for greater external validity. That is, there may be greater variability in the contexts where an educational innovation is examined and smaller effect size of an intervention, but when established, educators have greater assurance that any effect that is detected has greater consistency across the contexts of delivery. The messy complex nature of education and variability across curricula make practical trials well suited for evaluating the effects of curricular changes. This approach can complement more established program evaluation strategies within educators’ evaluation tool kits. Rather than focusing on whether an intervention changed educational outcomes (e.g. Kirkpatrick), why it worked, or identifying unintended outcomes -- practical trials can ensure curricular changes are feasible and acceptable.

A key feature of practical trials is the use of non-inferiority and equivalence study designs to make comparisons [13–15]. Rather than hypothesis testing for the superiority of one intervention compared to another, these methods aim to determine whether any difference between interventions is meaningful (not just significant) as determined by a minimally relevant difference set a priori. This minimally relevant difference is called the *non-inferiority margin*, which reflects the minimal acceptable difference between the two interventions that would be considered educationally significant. For example, if we determine a non-inferiority margin of 10-points on an exam and we find the mean difference between two interventions is less than 10-points, we would interpret the two interventions as non-inferior to one another. Alternatively, if there is a difference at or above this 10-points, we would interpret one of the interventions as superior. A benefit of this approach is the a priori clarity about what is and is not relevant for comparison. A practical trials approach requires determining whether any potential differences are of practical significance, which contrasts with mere statistical significance.

### The Need for Validity Evidence

Adapting practical trials to evaluate curricular change successfully will require strong validity evidence. Our confidence in our claims to adopt, expand, or end a curricular change should be determined by the quality of our validity evidence that supports the inferences we make from our evaluation data. Validity frameworks can be used to guide educators in establishing validity evidence with evaluation data by organizing validity evidence into different types and offering recommendations for how to gauge their strengths (or weaknesses) [16–18]. While practical trials and applying validity frameworks to improve the decision-making from assessment and evaluation data are not new, their combined application to evaluating curricular change with practical trials represents a novel innovation. Data obtained from conducting practical trials then, can benefit from validity evidence that supports their specified interpretation and use. That is, we can confidently interpret our assessment data as representative of our construct of interest (e.g., clinical competence, professionalism, or communication skills) and that our intended use case is appropriate and justified (e.g., the curricular innovation or change can be maintained because it did not result in educationally significant differences in our outcomes of interest).

To ensure meaningful inferences are made from curricular comparisons, we explore non-inferiority methods as an innovative means to evaluate curricular changes and offer insights that others can use to help select appropriate measures and strategies that enhance the validity of their findings. Using our institutional data, we evaluate the impacts of a recent curricular change to the anatomy curriculum on student learning effectiveness. The change reduced anatomy laboratory time for learners, and we sought to evaluate whether these changes affected student learning from the curriculum in a meaningful way. We use this data as a worked example to illustrate the various sources of validity evidence that can be considered when making curricular comparisons. We also describe how these considerations can refine our approach to curricular evaluations by exploring how our findings and interpretations can be altered by addressing these threats to validity or not. Namely, we explore meaningful comparisons between cohorts after a curriculum change can be affected by controlling for differences in exam difficulty and measures of student ability.

## Methods

### Participants and Setting

We retrospectively examined two cohorts of first- and second-year medical students (graduating classes of 2022 and 2025) across the three campuses of the University of Illinois College of Medicine (UICOM). After the pandemic, UICOM significantly reduced in-person anatomy lab time by roughly one-third (38 h). We were interested in evaluating the potential impacts of this change. Hence, we compared two cohorts’ summative exam performances on content from across the curriculum to determine whether recent changes in our curricular structure after the pandemic resulted in changes to students’ medical knowledge.

The curriculum prior to the changes included 108 h allocated to in-person anatomy labs featuring cadaver dissections. As a result of the pandemic, in 2020, most anatomy labs were taught online using faculty-developed computer-assisted learning modules and synchronous online tutorials. In 2021, the anatomy curriculum was further refined to reintroduce in-person anatomy labs that were complemented by the online computer-assisted learning modules. The refined curriculum included a total of 70 h allocated to anatomy labs for the graduating class of 2024; a significant reduction in student preparation and contact hours for these in-person labs (38 h).

To evaluate the effects of these changes, we selected cohorts from before (graduating class of 2022) and after (graduating class of 2025) the curricular change at time points where we considered the curriculum was stable. One cohort (class of 2022) experienced the anatomy curriculum before adjustments or prominent curricular changes (before the pandemic). The other cohort (class of 2025) experienced the modified curriculum a year after its original implementation, reducing potential confounding effects due to student and faculty adjustment or increased motivation and engagement due to the novelty of the change (after the pandemic).

### Measures

The 18-month long pre-clinical UICOM curriculum is organized into 8 organ-based blocks of content; each block ends with a summative exam. For cohort comparison, we focused on students’ cumulative exam scores from summative exams for four blocks of the curriculum (Block 3 to 6). We selected all exam items from these Blocks, which included musculoskeletal (Block 3), cardio-respiratory system (Block 4), alimentary and urinary systems (Block 5), and head and neck anatomy (Block 6). These blocks were selected because they had mandatory dissections (i.e., had in-person anatomy lab time reduced) and were hypothesized to be most affected by the curricular change.

These summative block exams also have strong validity evidence based on inferences of scoring, generalization, extrapolation, and implications [16]. The scoring and generalization inferences of the exams are robust, with exams scores demonstrating high internal consistency within and between each other. The validity of generalization and implications inferences from these exams are supported by their predictive value for future clinical performance, as evidenced by their high correlation (*r* >0.80) with USMLE Step 1 scores (when they were offered) and of pass/fail. 

Overall test cut-off scores, or minimum passing level (MPL), were used to determine the difficulty level of exams administered to the two cohorts. At our institution, a minimum of three subject matter experts from each discipline review each exam item to gauge item difficulty for each test item and assign Angoff ratings of 0, 0.5, or 1 based on the percent likelihood that a borderline student would correctly answer the item [19]. Overall exam difficulty was determined by averaging the Angoff scores for each test item. This rigorous test construction process ensures appropriate content and difficulty of item scores; and represents a source of validity evidence for scoring inferences made from test item scores. ﻿We also collected data about each cohort’s general academic ability and test difficulty using MCAT scores and GPA at admission, which were used as ﻿potential ﻿covariates in our analyses. 

### Analyses

#### Non-Inferiority Design

Using a non-inferiority study design, we employed multiple linear regression to compare the cumulative exam scores between the two cohorts. We established an a priori non-inferiority margin of 10% points based on students’ average summative scores from Block exams 3 to 6. We selected this value based on prior non-inferiority studies of clinical learning [20–22], our experience in exam development and grading at UICOM, and the assumption that a decline within 10% points would not substantially alter academic standards or impact clerkship training. In line with non-inferiority methods, we used a confidence interval for the mean differences in average exam scores to determine non-inferiority or practical difference (i.e., one cohort had superior scores). We selected a 99% confidence interval (a larger confidence interval) to add additional rigor to our interpretation [23]. Thus, the cohorts were considered non-inferior—indicating no practical difference—if the lower bound of the 99% confidence interval for the mean difference in average exam scores fell within this 10-percentage point margin. Conversely, if the confidence interval exceeded 10% points, we would conclude that a significant, practical difference exists, favoring one cohort over the other.

#### Adjusted Comparisons

To examine whether our interpretation of non-inferiority was affected by differences between cohorts, we ran the same regression analyses after controlling for differences in student ability and adjusting scores due to exam difficulty. For student ability, we used students’ science GPAs and MCAT scores as proxies for academic ability. The science GPA represents students’ cumulative GPAs from their undergrads restricted to only their science courses, a strategy adopted by UICOM to more precisely capture ability related to the content taught in the medical curriculum. The MCAT was selected due to its well demonstrated predictive value for future academic performance.

For exam difficulty, we corrected test scores between the two cohorts using the exam Angoff scores for each summative exams. Specifically, we computed the average Angoff scores (exam MPL) for each summative exam, then averaged them to determine a cumulative MPL value for all block exams experienced by both cohorts. The difference in the cumulative exam MPL between the cohorts was used to generate a difficulty-adjusted exam score that could be used to compare cohorts. That is, if one set of exams had a cumulative MPL that was lower by 5% (and was thus more difficult), the average raw exam scores for these learners would have 5% points added to their scores to produce a difficulty-adjusted exam score. Angoff scores were selected due to the availability of the data and its ability to correct for overall exam difficulty.

The Institutional Review Board of the University of Illinois at Chicago reviewed the study (STUDY2023-0082-MOD004) and determined that the study qualified for exemption.

## Results

We collected exam data from 304 to 300 students from the graduating classes of 2022 and 2025 respectively. We noted minor differences in the exam length for each of the 4 summative exams. Using our average Angoff scores for the 4 summative exams for each cohort, we found students in the 2025 cohort had easier exams (MPL_2022_ = 58.31, MPL_2025_ = 63.46). From the cumulative MPL, we developed a corrective value of 5.15% using the difference in MPLs between the cohorts. This corrective value was added to the cumulative exam scores of students from the 2022 cohort to create a difficulty-adjusted exam score.

To determine whether the cohort comparisons were relevant, we present our results in a series of regression models before and after adjustments based on test difficulty and academic ability. Summary data for the exams and cohort data are provided in Tables 1 and 2 respectively.

**Table 1:** Cohort exam characteristics and minimum passing levels

	Number of Questions in Exam		Minimum Passing Level (MPL - Average Angoff Score x 100%)	
Summative Exam	Class of 2022	Class of 2025	Class of 2022	Class of 2025
Musculoskeletal	104	99	56.65	63.34
Cardiovascular Respiratory	111	110	54.45	62.71
Alimentary and Urinary Systems	107	109	62.58	65.36
Head and Neck	108	107	59.97	62.39
Total	430	425	Cumulative MPL: 58.31	Cumulative MPL: 63.46

**Table 2.** Summary of Cohort Differences in GPA, MCAT, and Exam Scores. Values reported as means (standard error of the mean) unless specified otherwise.

	Class of 2022	Class of 2025	Mean Difference (99% Confidence Interval)
GPA	3.62 (0.02)	3.57 (0.02)	-
MCAT	511.91 (0.36)	510.16 (0.39)	-
Average Raw Exam Scores (%)	76.52 (0.43)	76.65 (0.47)	0.13 (−1.77, 1.50)
Difficulty-Adjusted Exam Scores (%)	81.62 (0.43)	76.65 (0.47)	−4.97* (−6.66, −3.33)
Difficulty-Adjusted Exam Scores Accounting for MCAT and GPA (%)	81.18 (0.40)	76.91 (0.41)	−4.27* (−5.77, −2.77)

* denotes a significant difference < 0.01

### Raw Exam Scores vs. Difficult-Adjusted Exam Scores

We found no significant difference between the cohorts in raw cumulative exam scores (Mean difference = 0.13%), *t* (602) = − 0.21, *p* = 0.836. This would suggest there are no significant differences between cohorts, and further, that this difference was practically small (less than 1%). However, using our difficult-adjusted exam scores, we found that students in the 2025 cohort scored significantly lower on their exams by 4.97%, *t* (602) =−7.985, *p* < 0.001, 99% CI [−6.66, −3.33]. Rather than rely on this statistical significance, we examined the 99% confidence intervals of the mean difference to determine its practical significance. Based on our a priori non-inferiority margin of 10%, we deemed these two cohorts’ difficulty-adjusted exam scores as non-inferior and thus inferred that the curricular change resulted in no practical difference in scores.

### Accounting for Cohort Differences in Ability (MCAT and GPA)

Including MCAT and GPA scores in our regression, we see a significant mean difference of 4.27%, *t* (583) = −7.37, *p* < 0.001, 99% CI [−5.77, −2.77]. Based again on our a priori non-inferiority margin of 10%, we interpret the curricular change the 2025 cohort of students experienced to be non-inferior to the experience of the 2022 cohort of students.

## Discussion

This study demonstrates how the validity of curricular comparisons can be improved using practical trials and by careful consideration of potential threats to validity. We found that reduced anatomy time in our curriculum (specifically in-person cadaver dissection) resulted in a significant but non-concerning change in our student exam scores. Our results provide a worked example of how curricular changes may impact student learning between two cohorts, and how different methodological decisions can lead different interpretations and conclusions. We describe these data, interpretations, and offer recommendations to support the validity of curricular comparisons below.

### Naive vs. Nuanced Curricular Comparisons

Despite the exams being on the same content the exams were different. We see from Table 1 that the cohorts had small differences in the number of items on each block exam (e.g., content area) and in their average difficulty. We found an average difference in cumulative exam difficulty of 5.15% points between the two cohorts. This finding highlights the need to investigate exams items and difficulty to establish (and potentially account for) comparability between cohorts, even for the “same” exams.

In comparing the raw average exam scores of the two cohorts directly, we observed a difference of roughly one-tenth of a percent between the cohorts that was nonsignificant. Taken at face value, we may be tempted to infer that there was no difference between our two cohorts. The curricular change would be vaunted as a success, concluding that reductions to dissection had no ill effect on student medical expertise (as measured by our summative exam scores, which were highly predictive of national licensing exam performance). However, when we accounted for exam difficulty between the two cohorts using average Angoff scores, we observed a significant difference of 4.97%; a difference with potential practical relevance.

While we may readily dismiss a nonsignificant difference of 0.13% in raw exam scores between cohorts, we may be less confident doing so for the 4.97% difference observed with our difficulty-adjusted exam scores. Using the non-inferiority approach offers additional validity evidence to help increase our confidence in our interpretation. Namely, establishing a non-inferiority margin, if done a priori, helps considerably with interpreting whether a difference is of practical significance. Simply put, if done well, it eliminates second-guessing from the equation, and can disincentivize "p-hacking" to find or remove effects by adjusting our non-inferiority margin post-hoc. Since we determined that a 10% difference was our minimally acceptable educational difference (and provided our rationale for doing so), we can confidently interpret this difference as practically insignificant. Our curricula for the class of 2025 are non-inferiority, at least based on our explicitly stated intents and purposes, because the 99% confidence interval of our mean score difference was between − 6.66% and − 3.33%; and did not exceed our non-inferiority margin of 10%.

We also observed more subtle changes when accounting for MCAT and GPA scores. We noted a decrease in our mean difference to 4.27% with a 99% confidence interval from − 5.77% to −2.77%. While this did not affect our final interpretation of our comparison, this change illustrates the importance of including measures of students’ ability. If our non-inferiority margin were, for example, 6% or 5%, we would have arrived at two vastly different interpretations of our difference, which could have then led to stark differences in curricular decision-making. In the 6% scenario, we would have similarly declared the two cohorts as non-inferior, and in the latter 5% scenario, we would have declared the new cohort (class of 2025) as meaningfully inferior to the previous cohort. The same analyses with two highly different interpretations.

All in all, though we detected real differences (e.g., lower performance) due to the curricular change, the significant time and resource savings (38 h of curriculum team) suggest the changes are worth keeping or considering keeping. Even if our non-inferiority margin were different and we determined the change to be unacceptable, the freed-up resources could potentially be devoted back to enhance the anatomy curriculum – either by increasing in-person anatomy time, or exploring alternative means of instruction (e.g., more online modules). Either way, these findings offer new insights into considering the effects of curricular changes before they are made and/or analyzed, which includes collecting meaningful assessment data that can include efficiency measures (e.g., time and cost) in addition to learning effectiveness.

### Insights on Curriculum from Practical Trials

By conducting practical trials in real-world educational settings, we gain valuable insights into how this methodology can be improved while assessing the validity of our data. We demonstrate how strong validity evidence and exam practices, such as incorporating exam difficulty measures and selecting appropriate cohorts (during periods of curricular stability), can strengthen the validity of our interpretations. As well, we reveal the potential for covariates such as student ability to impact the confidence intervals and thus the interpretation of our findings. Our study also underscores the importance of determining one’s a priori non-inferiority margin, a critical factor that shapes how we make valid inferences from our findings. Taken together, our worked example of how to apply non-inferiority methods highlights a crucial lesson for curricular comparisons; it is not merely about what curriculum is more effective, but also what is more efficient at achieving comparable outcomes. Our reduction in in-person anatomy lab hours had a non-concerning effect on students’ grades, and created curricular time and savings in resources that could benefit other areas of the curriculum.

### Recommendations for Incorporating Practical Trials

Conducting meaningful practical trials of curricular change will require comparisons that consider the validity evidence that supports our interpretation and decision-making. Our analysis of our institutional data offers several methodological suggestions for other schools of medicine and health professions who aspire to more rigorously evaluate changes in their curricula using their own institutional data. First, apply practical trials (i.e., non-inferiority and equivalence study designs) to assess for practical impacts of curricular change. Second, select your assessment measures for alignment with your construct of interest and set an a priori value for your minimally relevant difference in this assessment. This includes selecting the timing of comparisons (select cohorts that represent more stable iterations of your curriculum) to avoid any transient effects of faculty and student enthusiasm and consistency in delivery. Third, ensure any relevant differences in assessments or student cohorts are accounted for in your analysis. In our case, we made sure to adjust our analyses to account for cohort differences in test difficulty and in student academic ability. These measures too require careful selection to ensure sufficient validity evidence in the final comparisons. Finally, pertaining to all previous suggestions, have discussions with curriculum stakeholders to help identify the appropriate assessments, non-inferiority margin (minimally acceptable educational difference), and cohort differences that should be controlled for. Some of these conversations and methods may require considering institutional processes for test development and data collection that will support future analyses and scholarship from these data. For example, test development protocols may need to be developed or refined to include processes to establish and maintain data regarding item difficulty.

While non-inferiority trials have been increasingly discussed in the education literature, they are limited by poor design. Though some studies do invoke non-inferiority or equivalence methods without defining their minimally relevant educational difference (required for determining one’s non-inferiority or equivalence margins) [24], or do so post-hoc [25], we strongly recommend that decisions be made *a priori*. This requires considering what constitutes a meaningful educational change, and what inferences educators would like to make from these data. The thoughtful use of practical trials will require tailoring measures to one’s local context. For example, considering different outcome measures (e.g., local anatomy practical exam scores) or precise confounding variables to control for (e.g., surgical shelf exam scores) that are both available and relevant to one’s curricular change and research question. Without clearly delineating the relevant measures and magnitudes of change, the validity of practical trials findings for curricular evaluation are weakened, and all analyses are complicated by a cornucopia of potential variables, comparisons, and interpretations. Trial registration is suggested as one potential strategy to help ensure rigor and transparency in reporting [12, 15]. With these pitfalls and potential solutions underscored, we believe researchers and educators will go a long way to improving the quality of their curricular evaluations and establishing more informed and robust decisions from their analyses.

### Limitations

A limitation of our findings is the single institutional approach. Given the complexities of curricular changes, we cannot assume that the decrease in cumulative exam scores across the cohorts was due solely to our changes in anatomy curriculum (as large as they may seem). Also, the magnitude of changes observed in our results are tied to our specific context at our institution. However, our aim of this study was not to generalize the notion that all reductions in anatomy lab time can be done without significant harm to medical students’ expertise of basic science knowledge taught in medical school. Rather, we demonstrate the value in critical consideration of cohort comparisons as a methodology to evaluate the effectiveness of curricular changes. Another limitation of our findings is the focus on learning as measured by students’ mastery of clinical knowledge on summative exams. We cannot say whether these changes had other impacts on the learning environment or other factors. However, we argue that the comparative methods we describe can be similarly applied to measure these other aspects of curriculum (e.g., student satisfaction). Relatedly, there may be other confounders that other researchers deem relevant to include in addition to measures of student ability and exam difficulty. In our study, we included these two constructs because we felt they would be relevant (and feasible) for other researchers to account for (student ability and exam difficulty). We believe these decisions are best left for researchers who understand the nuanced drivers and constraints within their own curricular context to determine.

## Conclusions

Practical trials offer new opportunities for schools and centers of health professions education to develop a more nuanced understanding of what changes are effective and efficient in their curricula and help leaders and educators make more informed decisions about how they approach curricular change and their evaluation. While non-inferiority designs are increasingly discussed in medical education research, their application to evaluating large scale medical curricular changes is rare and introduces threats to validity. Our study demonstrates how design decisions (e.g., cohort selection) and available student and exam data (e.g., Angoff scores) are critical to ensuring strong validity evidence of our assessments and thus our interpretations of curricular cohort comparisons. The innovative approach we recommend for applying practical trials can serve as a model for all educational institutions seeking more rigorous and nuanced understanding of the impacts of curricular innovations.
